# Supplementary figures and images for: Intraspecific Variation in the Placement of Campaniform Sensilla on the Wings of the Hawkmoth Manduca Sexta
Source: Integr Org Biol. 2024 Mar 13;6(1):obae007. doi: 10.1093/iob/obae007 (PMC11074993; doi:10.1093/iob/obae007)

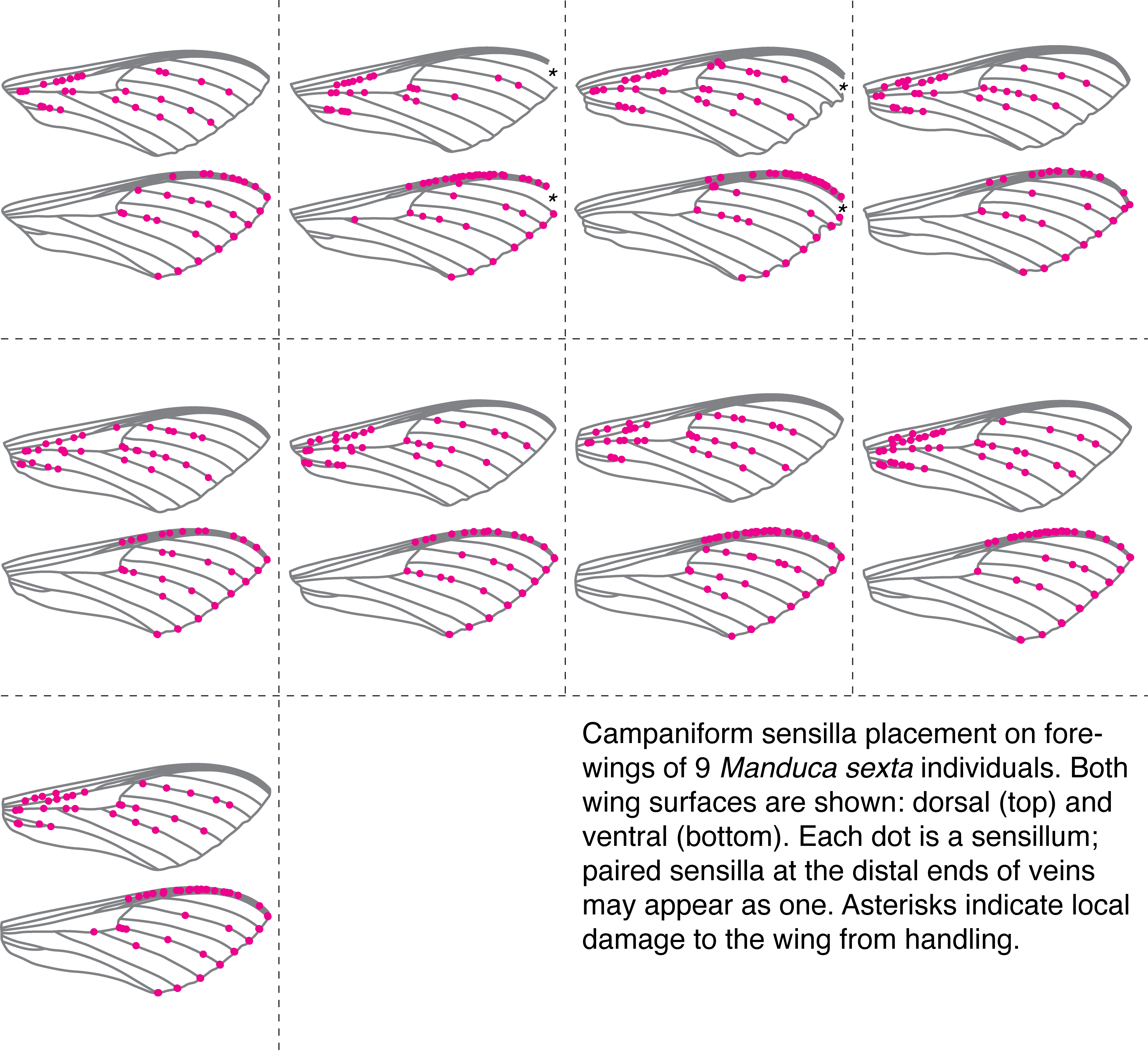

Supplement: obae007_Supplemental_Files [file obae007_supplemental_files.zip › SupplementalFigure1_Forewings.tif]

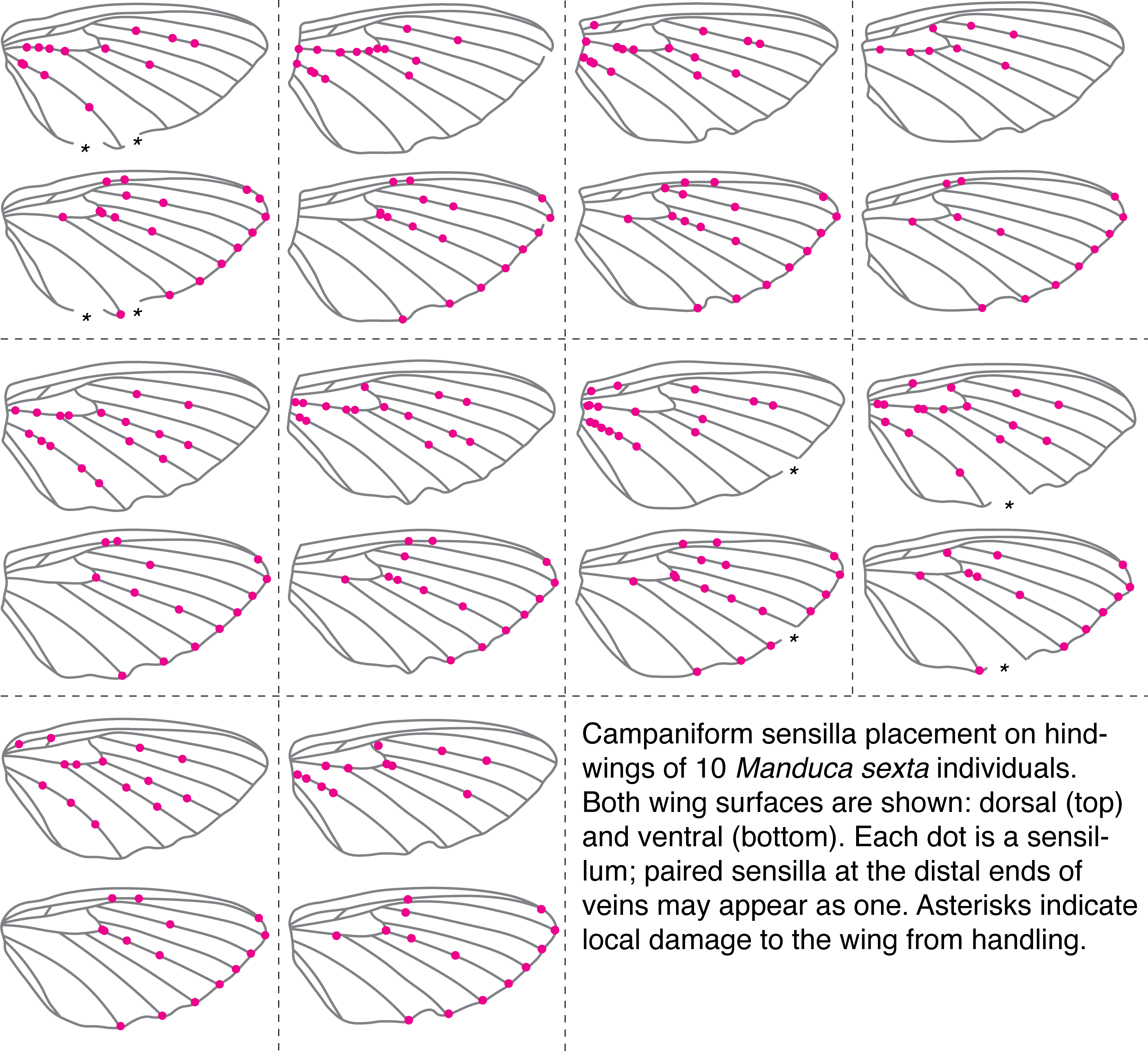

Supplement: obae007_Supplemental_Files [file obae007_supplemental_files.zip › SupplementalFigure2_Hindwings.tif]
